# Supplementary material for: Unravelling some factors affecting sexual reproduction in rock-specialist shrub: Insight from an endemic Daphne arbuscula (Thymelaeaceae)
Source: PLoS One. 2024 May 9;19(5):e0300819. doi: 10.1371/journal.pone.0300819 (PMC11081377; doi:10.1371/journal.pone.0300819)
Supplement: Attachment — Submitted filename: PONE-D-23-32886 - MS with comments and questions from reviewer.pdf [file pone.0300819.s002.pdf]

PLOS ONE

Unravelling some factors affecting sexual reproduction in rock-specialist shrub: insight from an endemic Daphne arbuscula (Thymelaeaceae)

--Manuscript Draft--

**Manuscript Number:**	PONE-D-23-32886
**Article Type:**	Research Article
**Full Title:**	Unravelling some factors affecting sexual reproduction in rock-specialist shrub: insight from an endemic Daphne arbuscula (Thymelaeaceae)
**Short Title:**	Factors affecting sexual reproduction in rocky endemic shrub
**Corresponding Author:**	Marek Slovak, Ph.D.<br>Slovak Academy of Sciences Institute of Botany: Botanicky ustav Slovenskej akademie vied<br>Bratislava, SLOVAKIA
**Keywords:**	The Carpathians; endemism; Europe; fruit production; RAD sequencing; sexual reproduction, vascular plants
**Abstract:**	The role of endemic species in global biodiversity is pivotal, and understanding their biology and ecology is imperative for their fitness and long-term survival, particularly in the face of ongoing climatic oscillations. Our primary goal was to investigate the sexual reproduction level of the endangered Western Carpathian endemic Daphne arbuscula (Thymelaeaceae), which inhabits extreme rocky habitats, and to comprehend the influence of specific factors on its reproductive success. We conducted the research across four populations, varying in size and environmental conditions. Over two years, we monitored flower and fruit production, analyzed genetic variability within and between populations, and studied pollination mechanisms. Daphne arbuscula proved to be a strict outcrosser, with significant variations in flower and fruit production among populations and seasons. The average fruit production percentage consistently remained below 50%, indicating challenges in sexual reproduction. Cold and harsh weather during the reproductive phase had a substantial negative impact on sexual reproduction efficacy, leading to decreased fruit production. Nevertheless, several individuals in sheltered microhabitats displayed significantly higher fruit production, ranging from 60% to 83%, emphasizing the critical role of microhabitat heterogeneity in sustaining sexual reproduction in this species. We found no pronounced differences in genetic diversity within or between populations, suggesting that genetic factors may not critically influence the reproductive success of this endemic species. The implications of our findings might be of paramount importance for the long-term survival of D. arbuscula and offer valuable insights for the development of effective conservation strategies for this species.
**Order of Authors:**	Zuzana Gajdošová
	Marek Šlenker
	Marek Svitok
	Gabriela Šrámková
	Drahoš Blanár
	Veronika Cetlová
	Jaromír Kučera
	Ingrid Turisová
	Peter Turis
	Marek Slovak, Ph.D.
**Additional Information:**	
**Question**	**Response**

Powered by Editorial Manager® and ProduXion Manager® from Aries Systems Corporation

**Financial Disclosure**<br><br>Enter a financial disclosure statement that describes the sources of funding for the work included in this submission. Review the submission guidelines for detailed requirements. View published research articles from *PLOS ONE* for specific examples.<br><br>This statement is required for submission and **will appear in the published article** if the submission is accepted. Please make sure it is accurate.<br><br>**Funded studies**<br>Enter a statement with the following details:<br>• Initials of the authors who received each award<br>• Grant numbers awarded to each author<br>• The full name of each funder<br>• URL of each funder website<br>• Did the sponsors or funders play any role in the study design, data collection and analysis, decision to publish, or preparation of the manuscript?<br><br>Did you receive funding for this work?	Yes
Please add funding details.<br>as follow-up to "**Financial Disclosure**<br><br>Enter a financial disclosure statement that describes the sources of funding for the work included in this submission. Review the submission guidelines for detailed requirements. View published research articles from *PLOS ONE* for specific examples.<br><br>This statement is required for submission and **will appear in the published article** if the submission is accepted. Please make sure it is accurate.	This work was supported by the Slovak Research and Development Agency (no. APVV-22-0365 to MS); by Scientific Grant Agency of the Ministry of Education, Science, Research and Sports of the Slovak Republic and the Slovak Academy of Sciences (no. VEGA 2/0098/22 to JK) and 'Conserving the endemic flora of the Carpathian Region', (to MS and JK) managed by the Institute of Botany of the Slovak Academy of Sciences. Computational resources were provided by the e-INFRA CZ project (ID: 90254), supported by the Ministry of Education, Youth, and Sports of the Czech Republic. M. Svitok was supported by the Operational Programme Integrated Infrastructure (OPII), funded by the ERDF (ITMS 313011T721). Funders did not play any role tudy design, data collection and analysis, decision to publish, or preparation of the manuscript.

Powered by Editorial Manager® and ProduXion Manager® from Aries Systems Corporation

**Funded studies**<br>Enter a statement with the following details:<br>• Initials of the authors who received each award<br>• Grant numbers awarded to each author<br>• The full name of each funder<br>• URL of each funder website<br>• Did the sponsors or funders play any role in the study design, data collection and analysis, decision to publish, or preparation of the manuscript?<br><br>Did you receive funding for this work?"	
Please select the country of your main research funder (please select carefully as in some cases this is used in fee calculation).<br>as follow-up to "**Financial Disclosure**<br><br>Enter a financial disclosure statement that describes the sources of funding for the work included in this submission. Review the submission guidelines for detailed requirements. View published research articles from *PLOS ONE* for specific examples.<br><br>This statement is required for submission and **will appear in the published article** if the submission is accepted. Please make sure it is accurate.<br><br>**Funded studies**<br>Enter a statement with the following details:<br>• Initials of the authors who received each award<br>• Grant numbers awarded to each author<br>• The full name of each funder<br>• URL of each funder website<br>• Did the sponsors or funders play any role in the study design, data collection and analysis, decision to publish, or preparation of the manuscript?<br><br>Did you receive funding for this work?"	SLOVAKIA - SK
**Competing Interests**	he authors have declared that no competing interests exist.

Powered by Editorial Manager® and ProduXion Manager® from Aries Systems Corporation

Use the instructions below to enter a competing interest statement for this submission. On behalf of all authors, disclose any competing interests that could be perceived to bias this work—acknowledging all financial support and any other relevant financial or non-financial competing interests.<br><br>This statement is required for submission and **will appear in the published article** if the submission is accepted. Please make sure it is accurate and that any funding sources listed in your Funding Information later in the submission form are also declared in your Financial Disclosure statement.<br><br>View published research articles from *PLOS ONE* for specific examples.<br><br>**NO authors have competing interests**<br><br>Enter: *The authors have declared that no competing interests exist.*<br><br>**Authors with competing interests**<br><br>Enter competing interest details beginning with this statement:<br><br>*I have read the journal's policy and the authors of this manuscript have the following competing interests: [insert competing interests here]*<br><br>* typeset	
**Ethics Statement**<br><br>Enter an ethics statement for this submission. This statement is required if the study involved:<br><br>• Human participants<br>• Human specimens or tissue<br>• Vertebrate animals or cephalopods<br>• Vertebrate embryos or tissues	1. Human Subject Research: not applicable<br>2. Animal Research: not applicable<br>3. Field Research: Permission for collection of D. arbuscula no. 11287/2021–9.1 (30/2021–rozkl.) provided by the Ministry of Environment of the Slovak Republic on November 8, 2021

• Field research<br><br>Write "N/A" if the submission does not require an ethics statement.<br><br>General guidance is provided below. Consult the submission guidelines for detailed instructions. **Make sure that all information entered here is included in the Methods section of the manuscript.**	

**Format for specific study types**<br><br>**Human Subject Research (involving human participants and/or tissue)**<br>• Give the name of the institutional review board or ethics committee that approved the study<br>• Include the approval number and/or a statement indicating approval of this research<br>• Indicate the form of consent obtained (written/oral) or the reason that consent was not obtained (e.g. the data were analyzed anonymously)<br><br>**Animal Research (involving vertebrate animals, embryos or tissues)**<br>• Provide the name of the Institutional Animal Care and Use Committee (IACUC) or other relevant ethics board that reviewed the study protocol, and indicate whether they approved this research or granted a formal waiver of ethical approval<br>• Include an approval number if one was obtained<br>• If the study involved *non-human primates*, add *additional details* about animal welfare and steps taken to ameliorate suffering<br>• If anesthesia, euthanasia, or any kind of animal sacrifice is part of the study, include briefly which substances and/or methods were applied<br><br>**Field Research**<br><br>Include the following details if this study involves the collection of plant, animal, or other materials from a natural setting:<br>• Field permit number<br>• Name of the institution or relevant body that granted permission	
**Data Availability**<br><br>Authors are required to make all data underlying the findings described fully available, without restriction, and from the time of publication. PLOS allows rare exceptions to address legal and ethical concerns. See the PLOS Data Policy and FAQ for detailed information.	Yes - all data are fully available without restriction

Powered by Editorial Manager® and ProduXion Manager® from Aries Systems Corporation

A Data Availability Statement describing where the data can be found is required at submission. Your answers to this question constitute the Data Availability Statement and **will be published in the article**, if accepted.<br><br>**Important:** Stating 'data available on request from the author' is not sufficient. If your data are only available upon request, select 'No' for the first question and explain your exceptional situation in the text box.<br><br>Do the authors confirm that all data underlying the findings described in their manuscript are fully available without restriction?	
**Describe where the data may be found in full sentences. If you are copying our sample text, replace any instances of XXX with the appropriate details.**<br><br>• If the data are **held or will be held in a public repository**, include URLs, accession numbers or DOIs. If this information will only be available after acceptance, indicate this by ticking the box below. For example: *All XXX files are available from the XXX database (accession number(s) XXX, XXX.*).<br>• If the data are all contained **within the manuscript and/or Supporting Information files**, enter the following: *All relevant data are within the manuscript and its Supporting Information files.*<br>• If neither of these applies but you are able to provide **details of access elsewhere**, with or without limitations, please do so. For example:<br><br>*Data cannot be shared publicly because of [XXX]. Data are available from the XXX Institutional Data Access / Ethics Committee (contact via XXX) for researchers who meet the criteria for access to confidential data.*<br><br>*The data underlying the results presented in the study are available from (include the name of the third party*	The data are provided as Supporting Information. The datasets generated for this study can be found in online repositories (https://www.ncbi.nlm.nih.gov/genbank/) with GenBank accession numbers OQ269432–OQ269447 for 16 ITS sequences original for this study and OQ632972–OQ632991 for 20 cpDNA sequences (rpl32–trnL region). The RADseq reads have been deposited in the NCBI Short Reads Archive (BioProject ID PRJNA989661, SRA: SRR25100387–SRR25100425).

Powered by Editorial Manager® and ProduXion Manager® from Aries Systems Corporation

and contact information or URL).<br>• This text is appropriate if the data are owned by a third party and authors do not have permission to share the data.<br><br>* typeset	
Additional data availability information:	

Manuscript_Gajdosova et al_2023 Click here to access/download;Manuscript;Gajdosova et al. 2023.docx

Unravelling some factors affecting sexual reproduction in rock-specialist shrub: insight from an endemic *Daphne arbuscula* (Thymelaeaceae)

Short title: Factors affecting sexual reproduction in rocky endemic shrub

Zuzana Gajdošová[1], Marek Šlenker[1], Marek Svitok[2,3], Gabriela Šrámková[4], Drahoš Blanár[5], Veronika Cetlová[1], Jaromír Kučera[1], Ingrid Turisová[6], Peter Turis[6], Marek Slovák[1, 4,*]

[1] Plant Sciences and Biodiversity Centre, Institute of Botany, Slovak Academy of Sciences, Bratislava, Slovak Republic

[2] Department of Biology and General Ecology, Technical University in Zvolen, Zvolen, Slovak Republic

[3] Department of Forest Ecology, Czech University of Life Sciences Prague, Suchdol, Praha 6, Czech Republic

[4] Department of Botany, Charles University, Praha, Czech Republic

[5] Muránska planina National Park Administration, Muráň, Slovak Republic

[6] Department of Biology and Ecology, Matej Bel University in Banská Bystrica, Banská Bystrica, Slovak Republic

* Corresponding author

E-mail: marek.slovak@savba.sk (MS)

Abstract

The role of endemic species in global biodiversity is pivotal, and understanding their biology and ecology is imperative for their fitness and long-term survival, particularly in the face of ongoing climatic oscillations. Our primary goal was to investigate the sexual reproduction level of the endangered Western Carpathian endemic *Daphne arbuscula* (Thymelaeaceae), which inhabits extreme rocky habitats, and to comprehend the influence of specific factors on its reproductive success. We conducted the research across four populations, varying in size and environmental conditions. Over two years, we monitored flower and fruit production, analyzed genetic variability within and between populations, and studied pollination mechanisms. *Daphne arbuscula* proved to be a strict outcrosser, with significant variations in flower and fruit production among populations and seasons. The average fruit production percentage consistently remained below 50%, indicating challenges in sexual reproduction. Cold and harsh weather during the reproductive phase had a substantial negative impact on sexual reproduction efficacy, leading to decreased fruit production. Nevertheless, several individuals in sheltered microhabitats displayed significantly higher fruit production, ranging from 60% to 83%, emphasizing the critical role of microhabitat heterogeneity in sustaining sexual reproduction in this species. We found no pronounced differences in genetic diversity within or between populations, suggesting that genetic factors may not critically influence the reproductive success of this endemic species. The implications of our findings might be of paramount importance for the long-term survival of *D. arbuscula* and offer valuable insights for the development of effective conservation strategies for this species.

Introduction

Endemism is widely recognized as a key factor in prioritising biodiversity conservation strategies [1, 2]. To ensure the effective protection of endemic plants, it is crucial to have a comprehensive understanding of their evolution, life history, biology, and ecology and to identify the factors and mechanisms that contribute to their fitness and long-term survival [1–7]. In this regard, understanding the limitations that hinder their colonization of broader distribution areas is of particular interest [6, 8–11]. Furthermore, numerous studies have demonstrated that endemic species often exhibit lower reproductive success or dispersal abilities compared to their widespread congeners [3, 4, 9, 11]. The mode of reproduction also has a significant impact on the genetic structure of populations, particularly in species with small and isolated populations [10–13]. For instance, the presence of selfing or increased clonality might lead to decreased genetic diversity within and between populations, genetic drift, and inbreeding depression [3, 6, 13–15]. However, dispersal ability might be strongly influenced by environmental conditions, and the phylogenetic context of the studied plants should also be taken into consideration [4, 6]. Nevertheless, it is crucial to bear in mind that not all endemic plant species face reproductive and dispersal challenges when generalizing these findings [3, 6].

This study focuses on *Daphne arbuscula* Čelak. (Thymelaeaceae), a narrow endemic plant occurring in the Muránska planina Mts in the Western Carpathians (S1 Fig) [16–18]. It is a long-lived, evergreen shrub that grows to a height of 10–30 cm, has coriaceous leaves, and bears bisexual, strongly fragrant flowers ranging in colour from pink to purple, rarely even white (Fig 1A) [16–19]. The species is diploid, with a chromosome count of $2n = 2x = 18$ [20–22]. It is referred to as an outcrosser and is predominantly pollinated by various insect species [23, 24]. The flowering period of this species is relatively short, spanning two to three weeks

in May and/or June. Populations from colder localities often exhibit delayed flowering, sometimes up to one month compared to populations from warmer sites [18, 25,26]. Fruits are one-seeded hard dry or fleshy drupes in brownish to yellow-green colours, often enclosed within marcescent hypanthium structures (Fig 1B) [17, 27]. The fruit production of *D. arbuscula* is reported to be low, and this could potentially have a negative impact on its fitness and survival [cf. 17, 23, 26].

Fig 1. *Daphne arbuscula* and studied sites in the Muránska planina Mts. (A) flowers; (B) fruits; (C) shrub with bagged inflorescences; (D) Poludnica; (E) Šiance; (F) Malá Stožka; (G) Veľká Stožka. Photo: Z. Gajdošová.

Daphne arbuscula serves as an ideal model for investigating sexual reproduction in endemics due to its unique ecological characteristics and habitat preferences. It is primarily a saxicolous plant that thrives in extreme habitats, particularly on relict limestone cliffs and rocks in the mid-altitudinal montane zone (Figs 1D–G) [16–18]. Although these habitats provide a diverse range of microclimatic conditions, most of them are susceptible to weather fluctuations. Compared to grassland or forest vegetation, open rocky habitats are more vulnerable to oscillations in temperature, droughts, a lack of protective snow cover, limited root space, intense UV radiation, wind exposure, and gravitational challenges [28–31]. Such extreme environmental conditions experienced during the reproductive phase of plants can negatively affect the formation and development of sexual organs and reproductive propagules, potentially leading to impaired sexual reproduction [32–36]. The populations of *D. arbuscula*, although limited to one mountain system, demonstrate significant niche diversity, specifically those residing at lower altitudes and facing south have typically drier and warmer climatic conditions, whereas those living at higher altitudes on the northern slopes experience harsher and wetter

conditions with a montane microclimate [17, 18]. All populations are naturally isolated by dense mixed forest vegetation, resulting in a forest-free 'island system' that is essentially unconnected [cf. 28]. These ecological configurations might have significant implications for the reproductive success of the species and can influence the genetic structure within and between populations. Furthermore, in certain cases, smaller populations with fewer individuals may deviate from major reproductive systems and favour autogamy or vegetative reproduction. Such shifts can significantly influence genetic variability, fitness, and the survival of the given species [37, 38]. Previous studies have provided fragmentary data on the biology of *D. arbuscula*, including its reproductive system [17, 20, 22–25, 27, 39]. In addition, these studies were conducted with small sample sizes and did not consider environmental variations among populations. As a result, our understanding of its reproductive strategies, fitness, and survival abilities remains limited.

The primary objective of this study is to explore the overall fruit production of *D. arbuscula* and test whether it is decreased as previously hypothesised [17, 23, 26]. Furthermore, the study aims to investigate the correlations between flower and fruit production and seasonal climatic patterns throughout the reproductive phase over a span of two years. It also entails analysing the genetic variability within and between populations, considering the size and ecological traits of the populations under examination. Additionally, we will examine whether *D. arbuscula* exhibits strict allogamy or if there are any indications of a transition towards some rarer sexual reproduction mechanism.

Material and methods

Sampling and design of the study

Four localities were selected to represent the entire range of ecological conditions within the distribution range of *D. arbuscula* in Muránska planina Mts (S1 Fig) [17]. Moreover, the presence of clonal growth in *D. arbuscula* [17] made it difficult to determine the exact number of genetically distinct individuals. To reduce bias in the final results and the interpretation of flower and fruit production, only individuals spatially separated by smaller rock formations or vegetation were considered, minimizing the chances of clone inclusion. Regarding population size, Poludnica is the largest locality, with over 1400 shrubs, followed by Veľká Stožka, which contains approximately 1100 plants. Malá Stožka has around 500 shrubs, and the smallest locality, Šiance, encompasses approximately 115 shrubs. The precise estimation of shrub numbers is challenging due to the highly inaccessible rocky terrain, making precise counting essentially impossible. The study sites, Poludnica and Šiance, predominantly face south and southeast and are situated at lower altitudes (approximately 800 m and 950 m above sea level). They represent a niche characterized by drier and warmer climatic conditions and are hence referred to as 'warm-microclimate' localities. The Šiance population is limited to small calcareous rock bodies, with most individuals located in open, sunny rocks (SI41–SI46). A few individuals (SI47–SI50) occur in shaded rock crevices beneath a mixed forest canopy on northern-oriented rocks. In contrast, the other pair of populations, Malá Stožka and Veľká Stožka, represent a niche with colder and more humid climatic conditions, predominantly facing north, and are located at higher altitudes (approximately 1150 and 1275 m above sea level). These localities are hence referred to as 'cold-microclimate' sites.

Ten individuals from each population were carefully chosen and marked with a unique metal marker for identification purposes. All the studied populations had ample size to ensure that the research did not adversely affect their fitness and viability. The experiment was conducted over two growing seasons, from April to July in both 2018 and 2019.

Breeding system experiments

Prior to conducting pollination and fruit production experiments, a preliminary test was carried out to assess the permeability of nylon bags with a mesh size of 0.5 mm. These bags were made from UV-limiting, breathable material to minimize the risk of flower and fruit degradation or microbial infestation. In the initial experiment during the 2017 season, one inflorescence with unopened buds was isolated from each individual in all localities. The presence of fruit would indicate successful pollination, either through small insects penetrating the mesh or self-pollination. Thrips (Thysanoptera), known to be important pollinators of *D. arbuscula* [17, 24], have a maximum size of 0.5 mm, making it possible for them to pass through the mesh. The experiment involving 40 inflorescences resulted in seven individuals producing eleven well-developed fruits, confirming the suitability of these sacks for testing fruit production. However, it was determined that they were unsuitable for self-compatibility tests, as at least some pollinators can come in contact with flowers.

To investigate self-compatibility, nylon bags with a smaller mesh size of less than 0.2 mm were utilized to prevent pollinators from transferring pollen from other inflorescences or plants. During the 2018 growing season, three inflorescences with unopened buds were isolated from each individual, totalling 120 shoots. Furthermore, to ensure selfing, assisted pollination experiments were conducted on a subset of analyzed plants (3–5 shrubs) at the Poludnica and Šiance localities. After the flowers opened, the nylon bags were removed from the isolated inflorescences, and the flowers were manually pollinated between flowers within the same inflorescence using a delicate, hand-made brush. Following pollination, the flowers were enclosed in nylon bags to prevent pollen transfer between flowers or from other plants via animal vectors. Bags from all experiments were collected once the fruits ripened.

Flower and fruit production

For each of the ten selected individuals in all populations, fifteen flowering shoots with one inflorescence were randomly chosen and isolated using nylon bags with a mesh size of approximately 0.5 mm (Fig 1C). A total of 150 shoots per population were isolated during each season. The isolation process took place in May and June, depending on local conditions (Fig 2A). At the time of isolation, the majority of flowers had reached the full-bloom stage for at least a few days to ensure the possibility of pollination. The number of flowers in each inflorescence was recorded, and the fruits were collected between June and July, once they had ripened and started dropping from the shoots. The number of fruits formed per branch was recorded.

Fig 2. The reproductive period of the studied populations of *Daphne arbuscula* and the corresponding environmental conditions. (A) flowering and fruiting periods based on our observations. Shifts in the flowering time of four individuals (SI47, SI48, SI49, SI50) inhabiting more shaded microhabitats from the Šiance population are depicted by thinner lines; (B) mean monthly temperature [°C] data for the Muránska planina Mts; (C) precipitation [mm] data for the Muránska planina Mts. Data extracted for the two studied seasons (2018–2019) and the long-term average (1994–2020) sourced from SolarGIS 2020 (Bratislava, Slovakia).

Previous studies have identified fruit polymorphism, which includes two distinct fruit morphotypes that differ in colour and mesocarp thickness, and are dispersed at approximately the same time [26, 27]. However, it remains uncertain whether these morphotypes represent true fruit polymorphism or different ontogenetic stages of the fruit ripening process. Despite seeds from the 'small-fruit' morphotype appearing to have lower viability compared to those from the 'big-fruit' morphotype, their viability still exceeds a threshold of 50% [27]. This means

that both morphotypes might contribute significantly to the sexual reproduction of this species, and therefore we decided not to differentiate the collected fruits by morphotype in this study.

Genetic analyses

We conducted a comprehensive genetic study on a species that lacked previous genetic data. Thus, to obtain the first insight into the genetic variation of the studied species, we employed traditional molecular markers, specifically the ITS1-5.8S-ITS2 region of ribosomal DNA (referred to as ITS; five individuals per population) and the *rpl32-trnL*$^{(UAG)}$ region of chloroplast DNA (referred to as *rpl32-trnL*; five individuals per population). For a more detailed investigation of population-level variability, we utilized double-digest RAD sequencing (dd RADseq; all 40 individuals).

Genomic DNA was extracted from young dried leaves of all sampled individuals using the Invisorb® Spin Plant Mini Kit (Invitek Molecular GmbH, Berlin, Germany) following the manufacturer's instructions, supplemented with PVP and 6 µL of RNase A. The extracted DNA was then purified using AMPure XP (Beckman Coulter Inc., Brea, California, USA).

ITS and cpDNA sequence amplification and processing

The ITS region was amplified using the ITS4 and ITS5 primers [40], while the *rpl32-trnL* region was amplified using the rpl32-F and trnL$^{(UAG)}$ primers [41]. PCR amplifications were carried out in a total volume of 10 µL, comprising 1 µL of template DNA, 0.25 U of DreamTaq DNA polymerase (Thermo Fisher Scientific Inc., Waltham, Massachusetts, USA), 1 µL of 10× DreamTaq buffer, 0.2 µL of dNTP Mix (10 mM each), and 0.2 µL of each forward and reverse primer (10 mM each). The PCR conditions for amplifying the ITS region were as follows: initial denaturation at 95°C for 3 min, followed by 30 cycles of denaturation at 95°C for 30 s, annealing at 51°C for 30 s, extension at 72°C for 1 min, and a final extension at 72°C for 10

min. For amplifying the *rpl32-trnL* region, the PCR conditions were as follows: initial denaturation at 80°C for 5 min, followed by 30 cycles of denaturation at 95°C for 1 min, annealing at 52°C for 1 min (with a 5% ramp to 65°C), extension at 65°C for 4 min, and a final extension at 65°C for 5 min.

PCR products were enzymatically purified before direct sequencing using a 1:2 mixture of Exonuclease I and FastAP Thermosensitive Alkaline Phosphatase (Thermo Fisher Scientific Inc., Waltham, Massachusetts, USA) or the NucleoSpin® Gel and PCR Clean-up kit (Macherey-Nagel GmbH & Co. KG, Germany). Sequencing was performed using the ABI 3730xl DNA analyzer at Eurofins Genomics Company (Konstanz, Germany). Sequences were edited and aligned using Geneious software R7.1.9 (Biomatters Ltd., Auckland, New Zealand).

dd RAD sequencing

All 40 individuals from four populations were genotyped for single nucleotide polymorphisms (SNPs) using the dd RADseq protocol of [42] with modifications [see 43]. The libraries were sequenced with 300 cycles (2×150 bp paired-end/PE reads) using the Illumina HiSeq platform (Macrogen Inc., Seoul, South Korea).

Raw RADseq data processing, variant calling, and filtration

The raw data processing, variant calling, and filtration were conducted as follows: the raw reads were demultiplexed using FASTX-Toolkit v. 0.0.14 [44], quality trimmed in Trimmomatic v. 0.36 [45], and filtered for PCR clones using the clumpify.sh script in BBTools v. 38.42 [46]. Since no reference genome was available, a de novo catalogue of RADseq loci was constructed using STACKS v. 2.53 [47, 48]. To determine the optimal Stacks parameters specific to our dataset and minimize erroneous splitting or lumping of loci, we followed the methods outlined in [49] and [50]. The final catalogue of RADseq loci was built using the following criteria: a

minimum coverage to identify a stack of 20× (-m 20), a maximum number of mismatches allowed between loci in each sample (-M) of 2, and a maximum number of differences among loci to be considered as orthologous (-n) of 2. The cluster_fast command (USEARCH v. 11) [51] was then utilized to combine similar sequences with more than 80% identity to create the non-redundant clusters. Subsequently, mapping on the non-redundant de novo catalogue of RADseq loci was performed using BWA v. 0.7.5 [52] and processed with Picard Tools v. 2.22.1 [53]. The GATK v. 3.7 [54] was employed to call and filter reliable variants. Only biallelic SNPs were selected, but variants matching the following criteria were removed: Quality by Depth (QD) < 2.0, FisherStrand (FS) > 60.0, RMS Mapping Quality (MQ) < 40.0, MQRankSum < -12.5, ReadPosRankSum < -8.0, SOR > 3.0. Furthermore, genotypes with a read depth lower than 8, samples with more than 40% missing genotypes, and variants with more than 30% missing genotypes were discarded.

RADseq data processing and analyses

To reveal between and within-population variability of the studied populations, multiple approaches were employed. Principal component analysis (PCA) was conducted using the adegenet package [55] in R 4.2.0 software [56]. To assess the potential genomic admixture of *D. arbuscula* individuals, the assignment of individuals to homogeneous clusters was inferred with ADMIXTURE 1.3 [57]. To mitigate linkage disequilibrium while considering the entire dataset, 100 datasets were generated by randomly selecting one SNP from each RAD locus. Subsequently, SNP data were further filtered by removing sites with a minor allele frequency <5.0% to reduce bias associated with erroneous genotypes and singletons [58]. For each dataset and K values ranging from 1 to 4, one replicate of ADMIXTURE was run using the AdmixPipe pipeline [59]. Results were summarized by Clumpak [60] with a similarity threshold of 0.9 for major clusters, aiding in the identification of potentially discordant modes within a single K

value. To visualize potential conflicting signals, a phylogenomic network was inferred using the neighbour-net algorithm in SplitsTree software [61] based on the Nei's genetic distance matrix calculated in the StAMPP R package [62].

Statistical analysis

Generalized linear mixed models (GLMM) [63] were utilized to investigate temporal and spatial differences in flower and fruit production of *D. arbuscula*. The models incorporated random effects of repeatedly measured individuals and fixed effects of time (years 2018 and 2019), population (Šiance, Malá Stožka, Veľká Stožka, and Poludnica), and their interaction. Initially, the number of flowers was fitted using a GLMM with a Poisson error distribution and a logarithmic link function. However, the model residuals displayed considerable underdispersion ($\varphi = 0.28$), which can lead to overestimated standard errors and misleading statistical inference [64]. To address this issue and minimize the type II error rate, we refitted the data using a flexible Conway-Maxwell-Poisson distribution, allowing us to model a wide range of dispersion levels [65]. No further violation of the model assumptions was detected in diagnostic plots of residuals.

The probability of fructification was calculated as a proportion of flowers that developed fruits out of all flowers recorded on a given plant. Initially, we modelled the data as binomial proportions with a logit link function. However, the binomial GLMM showed significant overdispersion ($\varphi = 1.33$), potentially leading to inflated type I error rates [66]. As a result, we refitted the data using a model with beta-binomial distribution [67] and a complementary log-log link function, efficiently handling the overdispersion. Likelihood ratio tests were employed to assess the significance of the model terms [63]. Whenever a significant overall effect was identified ($\alpha = 5\%$), we conducted a series of post hoc tests with Tukey-adjusted probabilities to compare the estimated marginal means [68]. Marginal pseudo-determination coefficients

were calculated for each GLMM to quantify the proportion of the total variation explained by the random and fixed effects (R2c) and fixed effects only (R2m) [69]. The analyses were performed in R v. 4.2.0 (56) using the libraries DHARMa [70], emmeans [71], ggplot2 [72], glmmTMB [73], and performance [74].

Impact of weather conditions on fruiting and flowering of *D. arbuscula*

To assess the influence of weather conditions on the flowering and fruiting of *D. arbuscula*, we collected climatic data for both studied years (2018 and 2019) and the long-term averages for the period between 1994 and 2020. The data included average monthly temperature (°C) and precipitation (mm) for the Muránska planina Mts area, extracted from SolarGIS 2020 (Bratislava, Slovakia).

Results

Tests for autogamy

Out of 120 inflorescences isolated by bags with a denser mesh (Ø < 0.2 mm), no fruits were developed. Fruit production was not observed in any of the isolated inflorescences, even in those with assisted self-pollination (S2 Table).

Interpopulation, intrapopulation, and seasonal variability in flower and fruit production

Flower production of *D. arbuscula* significantly varied among populations, although the differences were contingent upon the year of observation (time × population: $\chi 2 = 41.8$, df = 3, $p < 0.0001$). In 2018, the number of flowers produced across populations was relatively consistent (Fig 3A). However, the following year, a decline in flower production was observed across all populations, with the most significant reduction occurring in Malá Stožka, which diverged significantly from the flower number of Poludnica (t = 3.5, df = 1166, p = 0.0029) and Veľká Stožka population (t = 4.2, df = 1166, p = 0.0002). Although the overall model was highly significant ($\chi 2 = 508$, df = 8, $p < 0.0001$), the flower counts data were fitted relatively poorly (R2c = 0.09, R2m = 0.04).

Fig 3. Seasonal variability in sexual reproduction of the four studied populations of *Daphne arbuscula*. (A) the number of flowers in the inflorescence; (B) the probability of fructification. The estimates based on Generalized Linear Mixed Models (GLMM) are represented by circles, with their 95% confidence intervals displayed as error bars.

Fruit development took between 5 and 7 weeks after anthesis, depending on weather conditions. The fruit production was low, ranging from 8.97% to 48.49% across populations and both growing seasons (Table 1; Fig 3B). Significant differences in fruit production were observed among populations in both years (time × population: $\chi 2 = 258$, df = 3, $p < 0.0001$). In 2018, fructification was significantly more probable in the warm-microclimate locality Šiance than in the cold-microclimate populations of Malá Stožka (t = 4.54, df = 1166, $p < 0.0001$) and Veľká Stožka (t = 5.61, df = 1166, $p < 0.0001$; Fig 3B). Poludnica population also showed a significantly higher fructification rate than Veľká Stožka (t = 2.95, df = 1166, p = 0.017). However, in 2019, the opposite was true; the warm-microclimate population of Poludnica showed significantly lower fruit production than Šiance (t = 3.67, df = 1166, p =

0.0014) and the cold-microclimate populations of Malá Stožka (t = 5.35, df = 1166, p < 0.0001) and Veľká Stožka (t = 4.20, df = 1166, p = 0.0002). In Poludnica and Šiance, fruit production was approximately balanced between the two years, and the temporal pattern seems to match flower counts. In cold climate populations, considerable variability was observed in fruit production between the two years, which was less affected by flower production. The beta-binomial GLMM was highly significant ($\chi 2$ = 508, df = 8, p < 0.0001) and had good performance (R2c = 0.53, R2m = 0.30).

Table 1. Reproduction and population-genetic (single nucleotide polymorphism) characteristics of the studied population of *Daphne arbuscula.*

**Population**	**Poludnica**	**Šiance**	**Malá Stožka**	**Veľká Stožka**
Number of studied individuals	10	10	10	10
Population size estimate	1400	115	500	1100
**Flower and fruit production**				
2018				
Flower production	1187	1100	1121	1159
Fruit production	241	471	118	104
Fruit set [%]	20.30 (0–43.1)	42.82 (17.9–76.1)	10.53 (2.4–30.9)	8.97 (0–19.4)
2019				
Flower production	1055	909	794	1096
Fruit production	115	263	385	390
Fruit set [%]	10.90 (0–33.0)	28.93 (2.5–83.3)	48.49 (24.3–70.1)	35.58 (12.4–66.7)
**Population genetic data**				
Private alleles in the population	1958 (5.1%)	1323 (3.4%)	1431 (3.7%)	1481 (3.9%)
Average private alleles of individuals	155 (119–213)	107 (71–146)	107 (74–200)	116 (80–151)

The success rate of sexual reproduction at the individual level fluctuated more dramatically, ranging from 0% to 83% (S3 Table). A few individuals did not produce any fruits during the study period (e.g., PO16). In contrast, only a few individuals from populations Šiance, Malá Stožka, and a single individual from Veľká Stožka exhibited fruit production in excess of 50%

(S3 Table). During both seasons (S3 Table), certain individuals from locality Šiance, specifically SI47–SI49, exhibited remarkably high rates of fruit production (60–83%).

Genetic diversity

The genetic analysis using nuclear ITS and *rpl32-trnL* regions did not reveal any intraspecific diversity within and between the studied populations (GenBank accession numbers OQ269432–OQ269447 and OQ632972–OQ632991).

Regarding the RADseq analysis, a total of 8,394,840.59 (± 3,588,224.75 SD) high-quality reads were generated per sample, except for sample SI41, which yielded 533,938 reads. A de novo catalogue of RADseq loci was constructed using STACKS, resulting in 23,494 loci. After combining similar sequences with over 80% identity, the non-redundant catalogue comprised 17,485 loci. On average, 61.30% (±1.31% SD) of the reads were successfully mapped to this catalogue, resulting in a final mean coverage of 340.28× (±145.8 SD) for all samples, except SI41, which had a mean coverage of 20.6×. The raw dataset contained 38,368 SNPs. The sample SI41 was excluded from the final analysis due to high levels of missing data. Thus the final dataset consisted of 39 individuals. The RAD-seq reads have been deposited in the NCBI Short Reads Archive under the BioProject ID PRJNA989661 and SRA accession number SRR25100388-SRR25100425.

The genomic analyses consistently supported the presence of shallow population structure, indicating genetic distinctiveness among all four populations. PCA and ADMIXTURE analysis at K = 2 and K = 3 showed that Poludnica and Veľká Stožka populations are genetically closer, with separation only occurring at K = 4 (Figs 4A and B). Interestingly, even at K = 4, a minor cluster revealed their genetic similarity. In contrast, Malá Stožka was identified as the most divergent population among all the populations analyzed. Both ADMIXTURE analysis and the phylogenomic network generated by the neighbour-net algorithm suggested limited gene flow

between the analyzed populations (Fig 4C). The four populations exhibited different numbers of private alleles, ranging from 1323 (Šiance – 3.4% of total alleles) to 1958 (Poludnica – 5.1% of total alleles). No private fixed alleles were detected in the population. The low levels of between-population divergence were further supported by the pairwise population differentiation values (FST), which ranged from 0.0035 to 0.0079 (Table 2).

Fig 4. Population genetic analyses based on 38,368 single nucleotide polymorphisms and 39 individuals from four populations of *Daphne arbuscula*. (A) genetic clustering depicted by ADMIXTURE analysis at K = 2–4. Each individual is represented by a vertical bar, with colour proportionally indicating the segment assignment; (B) Principal component analysis (PCA) analysis. (C) Neighbour-Net as recovered by Splitstree. Population codes are as follows: MS – Malá Stožka, PO – Poludnica, SI – Šiance, VS – Veľká Stožka.

Table 2. Pairwise population F_{ST} estimates inferred from single nucleotide polymorphism data.

	**Poludnica**	**Šiance**	**Malá Stožka**	**Veľká Stožka**
**Poludnica**	–	0.0042	0.0063	0.0035
**Šiance**	–	–	0.0079	0.0055
**Malá Stožka**	–	–	–	0.0058

Climatic conditions during the sexual reproduction phase of *D. arbuscula*

The weather conditions during the flowering and fruiting periods in both studied seasons showed significant differences (refer to Figs 2B and C for a graphical representation of these patterns). In April and May 2018, when the southern warm-microclimate populations were in the flowering phase, the weather was characterized by dry and warm conditions. Subsequently,

in June 2018, during the blooming period of the cold-microclimate populations, temperatures were only slightly higher than the overall average, but there was a significant increase in rainfall. In July 2018, both temperature and rainfall exhibited more stable patterns and did not deviate from the overall averages. In contrast, 2019 presented a completely different scenario. During April and May 2019, the weather was much colder and rainier, with occasional episodes of frost and snowfall. However, in June, the weather became significantly drier and warmer, and this pattern remained stable throughout July.

Discussion

Influence of climatic conditions on seasonal and population fluctuations in *D. arbuscula* fruit formation

Our study revealed that overall sexual reproduction in *D. arbuscula* exhibits relatively low fruit production, not exceeding 50% at the population level. This trend is commonly observed in endemic species with restricted distribution ranges and small population sizes, especially when compared to their more widespread congeners [3, 4, 8–11, 75, 76].

Climatic conditions have the potential to impact plant reproduction, although species respond to unfavourable conditions in various ways [32, 35, 77]. We demonstrate that climatic conditions during the flowering and fruiting periods exert a significant influence on fruit production, surpassing factors such as genetic variability, location, and ecology of the studied populations [cf. 78, 79]. During this study, we observed two climatically contrasting years with divergent weather patterns during the reproductive phase of this species, which had a significant impact on fruit production (Figs 2B, C and 3B). The species flowers from late April to June, coinciding with substantial weather variability in Central European mountainous

regions [80]. In 2018, drier and warmer weather in April and May, followed by colder and wetter conditions in June, resulted in significantly higher fruit production in the earlier-flowering warm-microclimate populations (20.3 % in Poludnica; 42.82% in Šiance; Table 1) compared to the later-flowering cold-microclimate counterparts (10.53 % in Malá Stožka; 8.97 % in Veľká Stožka; Table 1). Conversely, in 2019, colder and wetter weather in April and May led to a remarkable decrease in fruit formation in warm-microclimate populations (10.9 % in Poludnica; 28.93 % in Šiance; Table 1), while drier and warmer conditions in June enhanced fruiting in cold-microclimate populations (48.49 % in Malá Stožka; 35.58 % in Veľká Stožka; Table 1). These findings indicate that cold and wet weather during the reproductive phase significantly diminishes fruit production. However, it appears that fruit production in populations residing in warmer environments demonstrated greater stability across both seasons, suggesting that they were less affected by weather fluctuations compared to populations with a cold microclimate (Fig 3B). This indicates that the sexual reproduction behaviour of populations residing in cold microclimates at north-oriented sites shows an increased vulnerability to between-year oscillations in a seasonal climate, which could exacerbate their susceptibility to ongoing trends in global climatic changes.

Indeed, harsh weather conditions, such as cold, high humidity, and strong winds, can damage gametes, flowers, and young fruits, leading to lower seed quality and fruit set [33, 35, 36]. [39] found that late frosts, prolonged droughts, and strong winds during fruit formation can cause tissue degeneration in *D. arbuscula* fruits. As climate change increases the incidence of extreme weather events, higher spring temperatures may result in earlier flowering, increasing the risk of exposure to late frosts and subsequent fruit damage [32, 34, 36].

Moreover, the flowering period of *D. arbuscula* shrubs is rather short and typically lasts up to two weeks [18, 25]. Previous field studies on *D. arbuscula* have identified potential insect pollinators in the vicinity of blooming shrubs under sunny and warm weather conditions, but

contrasting trends may emerge when hostile climatic conditions prevail [17, 23, 24]. Thus prolonged rainfall and low temperatures can further diminish insect pollinator activity, reducing the chances of successful pollination [17, 35, 81]. Such a deficiency in pollinators consistently leads to decreased fruit production in other *Daphne* species as well [82–84]. Nevertheless, rocky habitats exhibit high geomorphological heterogeneity, featuring diverse microsites characterized by distinct microclimates [85–87]. Within *D. arbuscula* populations, individual shrubs located just a few meters apart can significantly vary in flowering time and reproductive success due to microclimate variations. Microsites with hospitable microclimates might act as a buffer against harsh weather fluctuations, ensuring that some shrubs experience optimal conditions for sexual reproduction. This was clearly demonstrated in the Šiance population, where shrubs in open, sunny rock crevices (SI41–SI46) flowered 1–3 weeks earlier compared to those on north-oriented, more humid rocks shaded by mixed forests. As a result, shrubs from shaded microhabitats produced substantially more fruit during both seasons (60–83%; S3 Table) compared to those from open, sunny places (2.5–59%; S3 Table). Such microsites may thus play a crucial role as long-term reservoirs for sexual reproduction and the maintenance of genetic diversity within the species.

Genetic variability at the population level seems not to be associated with decreased sexual reproduction in *D. arbuscula*

Genetic mechanisms may contribute to seed and/or fruit abortion, leading to low fecundity and posing a risk to the long-term survival of plants [e.g., 77, 88–93]. Endemic species, often occurring in small and isolated populations, may be more vulnerable to reduced gene flow within and between populations, potentially experiencing genetic drift or inbreeding depression [10, 11, 13–15, 94, 95].

Daphne arbuscula is considered an ancient tertiary relict species that likely survived Pleistocene glaciations on the south-facing slopes of Muránska planina Mts [17, 18]. Our genetic analyses revealed a relatively shallow genetic structure among the studied populations (Fig 4). This finding, contrasting with the pronounced genetic structure observed in the Menorcan narrow endemic *D. rodriguezii* Texidor [96], suggests that the fragmentation of *D. arbuscula*'s distribution range in Muránska planina Mts, or alternatively, colonization from potential microrefugia, occurred more recently in the postglacial period. The genetic differentiation among populations is relatively weak, as indicated by the pairwise population differentiation (FST values), a low level of within-population private SNPs (ranging between 3.4% and 5.1% per population), and the absence of private fixed SNPs (see Results section and Tables 1 and 2). Moreover, the stronger genetic differentiation observed in the small populations of Malá Stožka and Šiance, as revealed by ADMIXTURE at K = 2–4 (Fig 4A), may be attributed to genetic drift rather than long-term isolation.

While variations in within-population genetic variability were observed in the studied populations, these differences do not seem to be clearly linked to fruit production. Specifically, the most abundant population, Poludnica, exhibited the highest level of genetic variability, yet reproductive success remained consistently low year after year. In contrast, the population of Malá Stožka, which is spatially the most isolated and the second smallest in size with lower within-population genetic variability, had the highest fruit set during the second year among all studied populations (Table 1; Fig 3B). These findings align with studies that have shown that low genetic diversity does not always result in reduced reproductive success [e.g., 97, 98]. Therefore, it can be concluded that the genetic variability of the analyzed populations does not seem to play a crucial role in the fruit production of this species.

The role of additional intrinsic and extrinsic factors in decreasing fruit production of *D. arbuscula*

The observed reduction in sexual reproduction in *D. arbuscula* is in line with the phylogenetic context, as other species within the genus *Daphne* also exhibit insufficient levels of fruit formation below 50% [17, 83, 99–103]. Nevertheless, there are exceptions, such as the widespread *Daphne mezereum* L., which has been reported to reach up to 72% fruit production [104]. The limitation in fruit production could be linked to the floral structure of the genus, wherein anthers are situated in the floral tube [19], facilitating the dispersal of numerous pollen grains on the stigma of the same flower, thus preventing adhesion of pollen from another plant [83, 84, 102, 103]. Consequently, for outcrossing species like *D. arbuscula*, numerous flowers remain unpollinated, or if pollinated, self-pollinated embryos degenerate during ripening [39, 83, 84, 102, 103]. Despite having high pollen viability (approximately 97%) in outcrossing *D. arbuscula*, indicating no significant issues with pollen development and fertility [20, 22], this morpho-anatomical pollination issue can lead to a decline in the number of embryos resulting from outcrossing pollination.

Another potential explanation for the reduced fruit production in studied species is the limited availability of mating partners within and between populations. Several studies have emphasized the significance of population size and density for the reproductive success of animal-pollinated plants [reviewed by 104]. The scarcity of mating partners is further compounded by self-incompatibility, which has also been demonstrated in *D. arbuscula* (S2 Table) [23, 25, 39], leading to the failure of viable seed formation from self-pollinated flowers [9, 15, 105].

Furthermore, the decreased fruit production could be attributed to potential shifts towards vegetative (clonal) reproduction [17, 39, 106], which might also significantly reduce the number of compatible mating partners [cf. 4, 8, 107–109]. However, the role of clonality in

this context can only be speculated upon since exploring clonality falls outside the scope of the present study. Further research, involving the identification of clones in the field and comparative examinations of sexual reproduction between clones and sexually derived individuals, will provide insights into the impact of clonality on the sexual reproduction of *D. arbuscula* [Gajdošová, work in progress].

Importantly, however, our analyses consistently revealed a decrease in fruit production across all studied populations, irrespective of population size. In fact, individuals with the highest fruit production were found even in smaller populations, such as Šiance and Malá Stožka (S3 Table). This suggests that the limited number of mating partners, for the reasons mentioned above, particularly in smaller populations, may not be the primary factor contributing to the observed reduction in sexual reproduction.

Conclusions

Our study enhanced the overall understanding of sexual reproduction in the narrow endemic shrub dwelling in extreme rocky habitats. We studied important factors influencing its sexual reproduction and uncovered the crucial impact of weather fluctuations on the sexual reproduction success of its populations. The species' high sensitivity to unpredictable climatic oscillations, particularly during the spring season, underscores the potential threats it may encounter in the face of climate change. However, we also reveal that the geomorphological heterogeneity of rocky habitats provides diverse microclimatic conditions, which could facilitate sexual reproduction and contribute to the long-term survival of this endemic species. The limited and fragmented distribution range of the studied species does not necessarily lead to significant genetic depletion in the populations. Despite their restricted geographic range, these populations maintain relatively stable genetic diversity, indicating the presence of mechanisms that preserve genetic variation within their habitat. Studying endemic species like

D. arbuscula provides valuable insights into their survival strategies and the challenges they face. By improving our knowledge of their biology and ecology, we can develop effective conservation measures to mitigate the potential effects of climate change and safeguard the unique components of biodiversity represented by species like *D. arbuscula.*

Acknowledgements

We are deeply thankful to Ivan Bryndza for his assistance in the field.

Data availability statement

The datasets generated for this study can be found in online repositories (https://www.ncbi.nlm.nih.gov/genbank/) with GenBank accession numbers OQ269432–OQ269447 for 16 ITS sequences original for this study and OQ632972–OQ632991 for 20 cpDNA sequences (*rpl32–trnL* region). The RADseq reads have been deposited in the NCBI Short Reads Archive (BioProject ID PRJNA989661, SRA: SRR25100387–SRR25100425).

References

1. Morrone JJ. Endemism. In: Fath B, editor. Encyclopedia of ecology. Oxford: Elsevier; 2008. 3:81–86.

2. Harrison S, Noss R. Endemism hotspots are linked to stable climatic refugia. Ann Bot. 2017; 119:207–214. doi: 10.1093/aob/mcw248.

3. Major J. Endemism: a botanical perspective. In: Myers AA, Giller P, editors. Analytical biogeography: an integrated approach to the study of animal and plant distributions. Dordrecht: Springer Science+Business Media; 1988. pp.117–146.

4. Lavergne S, Thompson JD, Garnier E, Debussche M. The biology and ecology of narrow endemic and widespread plants: a comparative study of trait variation in 20 congeneric pairs. Oikos. 2004; 107:505–518. doi: 10.1111/j.0030-1299.2004.13423.x.

5. Kier G, Kreft H, Lee TM, Jetz W, Ibisch PL, Nowicki C, et al. A global assessment of endemism and species richness across island and mainland regions. PNAS. 2009; 106:9322–9327. doi: 10.1073/pnas.0810306106.

6. Bruchmann I, Hobohm C. Factors that create and increase endemism. In: Hobohm C, editor. Endemism in vascular plants. Plant and vegetation 9. Dordrecht: Springer Science+Business Media; 20014. pp.51–68.

7. Breman E, Hurdu BI, Kliment J, Kobiv Y, Kučera J, Mráz P, et al. Conserving the endemic flora of the Carpathian Region: an international project to increase and share knowledge of the distribution, evolution and taxonomy of Carpathian endemics and to conserve endangered species. Plant Syst Evol. 2020; 306:59. doi: 10.1007/s00606-020-01685-5.

8. Kunin WE, Gaston KJ. The biology of rarity: patterns, causes and consequences. Trends Ecol Evol. 1993; 8:298–301. doi: 10.1016/0169-5347(93)90259-R.

9. Murray BR, Thrall PH, Gill AM, Nicotra AB. How plant life-history and ecological traits relate to species rarity and commonness at varying spatial scales. Austral Ecol. 2002; 27:291–310. doi: 10.1046/j.1442-9993.2002.01181.x.

10. Espeland EK, Emam TM. The value of structuring rarity: the seven types and links to reproductive ecology. Biodivers Conserv. 2011; 20:963–985. doi: 10.1007/s10531-011-0007-2.

11. Miller-Struttmann NE. Rarity and reproductive biology: habitat specialists reveal a complex relationship. Botany. 2013; 91:349–359. doi: 10.1139/cjb-2012-0274.

12. Weller SG. The relationship of rarity to plant reproductive biology. In: Bowles ML, Whelan CJ, editors. Restoration of endangered species: conceptual issues, planning, and implementation. New York: Cambridge University Press; 1994. pp. 90–117.

13. Leimu R, Mutikainen P, Koricheva J, Fischer M. How general are positive relationships between plant population size, fitness and genetic variation? J Ecol. 2006; 94:942–952. doi: 10.1111/j.1365-2745.2006.01150.x.

14. Kruckeberg AR, Rabinowitz D. Biological aspects of endemism in higher plants. Ann Rev Ecol Syst. 1985; 16:447–79. doi: 10.1146/annurev.es.16.110185.002311.

15. Vallejo-Marín M, Dorken ME, Barrett SCH. The ecological and evolutionary consequences of clonality for plant mating. Annu Rev Ecol Evol Syst. 2010; 41:193–213. doi: 10.1146/annurev.ecolsys.110308.120258.

16. Krippel E. Thymelaeaceae Juss. – Vrabcovníkovité. In: Bertová L, editor. Flóra Slovenska IV/4: Angiospermophytina, Dicotyledonopsida, Fabales-Convolvulales. Bratislava: Veda; 1988. pp.508–519.

17. Erdelská O, Turis P. Biology of *Daphne arbuscula* Čelak. (Thymelaeaceae). Biologia, Bratislava. 1995; 50:333–348.

18. Kochjarová J, Turis P, Feráková V. *Daphne arbuscula* Čelak. In: Čeřovský J, Feráková V, Holub J, Maglocký Š, Procházka F, Zezula A, et al., editors. Červená kniha ohrozených a vzácnych druhov rastlín a živočíchov SR a ČR 5 – Vyššie rastliny. Bratislava: Príroda; 1999. p. 456.

19. Halda JJ. The genus *Daphne*. Hronov: SEN Dobré; 2001.

20. Murín A. *Daphne arbuscula*. In: Májovský J, editor. Index of chromosome numbers of Slovakian flora (Part 6). Acta Fac Rerum Nat Univ Comen Bot; 1978. 26:1–42.

21. Murín A. Karyology of an endemic species *Daphne arbuscula* Čelak. Botanica. 1990; 37:35–40.

22. Gajdošová Z, Svitok M, Cetlová V, Mártonfiová L, Kučera J, Kolarčik V, et al. Incidence and evolutionary relevance of autotriploid cytotypes in a relict member of the genus *Daphne* (Thymelaeaceae). AoB Plants. 2023; 15: plad056. doi: 10.1093/aobpla/plad056.

23. Erdelská O, Petušík J, Pelikán V. Vývin semien lykovca muránskeho. Biologia, Bratislava. 1989; 44:13–19.

24. Turis P, Smetana V. Príspevok k poznaniu interakcií medzi hmyzom a lykovcom muránskym. In: Uhrin M, edtor. Výskum a ochrana prírody Muránskej planiny. Revúca: Správa CHKO Muránska planina; 1997. pp. 71–74.

25. Erdelská O. The development and degeneration of ovules of *Daphne arbuscula*. In Benčaťová B, Hrivnák R, editors. Rastliny a človek. Zvolen: Technická univerzita; 1998. pp. 37–40.

26. Gajdošová Z. Reproduction, genetic and caryologic variability of *Daphne arbuscula* Čelak. (Thymelaeaceae). M.Sc. Thesis, Matej Bel University in Banská Bystrica. 2020. Available from: https://opac.crzp.sk/?fn=detailBiblioForm&sid=EAE7EF6B68A1C5264CC51446D593

27. Di Sacco A, Gajdošová Z, Slovák M, Turisová I, Turis P, Kučera J, et al. Seed germination behaviour of the narrow endemic *Daphne arbuscula* (Thymelaeaceae), compared to the more widespread *Daphne cneorum.* Fol Geobot. 2021; 56:13–25. doi: 10.1007/s12224-021-09389-5.

28. Baskin JM, Baskin CC. Endemism in rock outcrop plant communities of unglaciated Eastern United States: An evaluation of the roles of the edaphic, genetic and light factors. J Biogeogr. 1988; 15:829–840. doi: 10.2307/2845343.

29. Médail F, Verlaque R. Ecological characteristics and rarity of endemic plants from Southeast France and Corsica: implications for biodiversity conservation. Biol Conserv. 1997; 80: 369–381. doi: 10.1016/S0006-3207(96)00055-9.

30. Lavergne S, Garnier E, Debussche M. Do rock endemic and widespread plant species differ under the leaf–height–seed plant ecology strategy scheme? Ecol Lett. 2003; 6: 398–404. doi: 10.1046/j.1461-0248.2003.00456.x.

31. Matthes U, Larson DW. Microsite and climatic controls of tree population dynamics: an 18-year study on cliffs. J Ecol. 2006; 94:402–414. doi: 10.1111/j.1365-2745.2005.01083.x.

32. Hedhly A, Hormaza JI, Herrero M. Global warming and sexual plant reproduction. Trends Plant Sci. 2009; 14:30–36. doi: 10.1016/j.tplants.2008.11.001.

33. Zinn KE, Tunc-Ozdemir M, Harper JF. Temperature stress and plant sexual reproduction: uncovering the weakest links. J Exp Biol. 2010; 61:1959–1968. doi: 10.1093/jxb/erq053.

34. Vitasse Y, Schneider LA, Rixen C, Christen D, Rebetez M. Increase in the risk of exposure of forest and fruit trees to spring frosts at higher elevations in Switzerland over the last four decades. Agric For Meteorol. 2018; 248:60–69. doi: 10.1016/j.agrformet.2017.09.005.

35. Goszka AR, Snell RS. Seed quality and seed quantity in red maple depends on weather and individual tree characteristics. Ecol Evol. 2020; 10:13109–13121. doi: 10.1002/ece3.6900.

36. Park IW, Ramirez-Parada T, Mazer SJ. Advancing frost dates have reduced frost risk among most North American angiosperms since 1980. Glob Change Biol. 2020; 27:165–176. doi: 10.1111/gcb.15380.

37. Eckert CG, Kalisz S, Geber MA, Sargent R, Elle E, Cheptou PO, et al. Plant mating systems in a changing world. Trends Ecol Evol. 2010; 25:35–43. doi: 10.1016/j.tree.2009.06.013.

38. Pellegrino G, Bellusci F, Palermo AM. Effects of population structure on pollen flow, clonality rates and reproductive success in fragmented *Serapias lingua* populations. BMC Plant Biol. 2015; 15:222. doi: 10.1186/s12870-015-0600-8.

39. Erdelská O. Successive tissue degeneration in unfertilized ovules of *Daphne arbuscula*. Acta Biol Crac. 1999; 41:163–167.

40. White TJ, Bruns TD, Lee SB, Taylor JW. Amplification and direct sequencing of fungal ribosomal RNA genes for phylogenetics. In: Innis MA, Gelfand DH, Sninsky JJ, White TJ, editors. PCR Protocol: a guide to methods and applications. San Diego: Academic Press; 1990. pp. 315–322.

41. Shaw J, Lickey EB, Schilling EE, Small RL. Comparison of whole chloroplast genome sequences to choose noncoding regions for phylogenetic studies in angiosperms: the tortoise and the hare III. Am J Bot. 2007; 94:275–288. doi: 10.3732/ajb.94.3.275.

42. Arnold B, Kim ST, Bomblies K. Single geographic origin of a widespread autotetraploid *Arabidopsis arenosa* lineage followed by interploidy admixture. Mol Biol Evol. 2015; 32:1382–1395. doi: 10.1093/molbev/msv089.

43. Knotek A, Konečná V, Wos G, Požárová D, Šrámková G, Bohutínská M, et al. Parallel alpine differentiation in *Arabidopsis arenosa*. Front Plant Sci. 2020; 11:561526. doi: 10.3389/fpls.2020.561526.

44. Hannon GJ. 2010. FASTX-Toolkit. Version 0.0.14 [software]. 2010. [cited 2023 Sep 28]. Available from: http://hannonlab.cshl.edu/fastx_toolkit.

45. Bolger AM, Lohse M, Usadel B. Trimmomatic: a flexible trimmer for Illumina sequence data. Bioinformatics. 2014; 30:2114–2120. doi: 10.1093/bioinformatics/btu170.

46. Bushnell, B. BBTools. Version 38.42 [software]. 2014. [cited 2023 Sep 28]. Available from: https://jgi.doe.gov/data-and-tools/bbtools.

47. Catchen J, Amores A, Hohenlohe P, Cresko W, Postlethwait J. Stacks: building and genotyping loci de novo from short-read sequences. G3: Genes Genomes Genet. 2011; 1:171–182. doi: 10.1534/g3.111.000240.

48. Catchen J, Hohenlohe P, Bassham S, Amores A, Cresko W. Stacks: an analysis tool set for population genomics. Mol Ecol. 2013; 22:3124–3140. doi: 10.1111/mec.12354.

49. Paris JR, Stevens JR, Catchen JM. Lost in parameter space: a road map for Stacks. Methods in Ecol Evol. 2017; 8:1360–1373. doi: 10.1111/2041-210X.12775.

50. Rivera-Colón AG, Catchen J. Population genomics analysis with RAD, reprised: Stacks 2. Methods Mol Biol. 2022; 2498:99–149. doi: 10.1007/978-1-0716-2313-8_7.

51. Edgar RC. Search and clustering orders of magnitude faster than BLAST. Bioinformatics. 2010; 26:2460–2461. doi: 10.1093/bioinformatics/btq461.

52. Li H, Durbin R. Fast and accurate short read alignment with Burrows-Wheeler Transform. Bioinformatics. 2009; 25: 1754–1760. doi: 10.1093/bioinformatics/btp324.

53. Picard Tools. Version 2.22.1 [software]. [cited 2023 Sep 28]. Available from: https://broadinstitute.github.io/picard/

54. McKenna A, Hanna M, Banks E, Sivachenko A, Cibulskis K, Kernytsky A, et al. The genome analysis toolkit: a MapReduce framework for analyzing next-generation DNA sequencing data. Genome Res. 2010; 20:1297–1303. doi: 10.1101/gr.107524.110.

55. Jombart T. adegenet: a R package for the multivariate analysis of genetic markers. Bioinformatics. 2008; 24:1403–1405. doi: 10.1093/bioinformatics/btn129.

56. R Core Team. R: a language and environment for statistical computing. Vienna: R Foundation for Statistical Computing; 2022. [cited 2023 Sep 28]. Available from: https://www.R-project.org/

57. Alexander DH, Novembre J, Lange K. Fast model-based estimation of ancestry in unrelated individuals. Genome Res. 2009; 19:1655–1664. doi: 10.1101/gr.094052.109.

58. Linck E, Battey CJ. Minor allele frequency thresholds strongly affect population structure inference with genomic data sets. Mol Ecol Resour. 2019; 19:639–647. doi: 10.1111/1755-0998.12995.

59. Mussmann SM, Douglas MR, Chafin TK, Douglas ME. AdmixPipe: population analyses in Admixture for non-model organisms. BMC Bioinform. 2020; 21:1–9. doi: 10.1186/s12859-020-03701-4.

60. Kopelman NM, Mayzel J, Jakobsson M, Rosenberg NA, Mayrose I. Clumpak: a program for identifying clustering modes and packaging population structure inferences across K. Mol Ecol Resour. 2015; 15:1179–1191. doi: 10.1111/1755-0998.12387.

61. Huson DH, Bryant D. Application of phylogenetic networks in evolutionary studies. Mol Biol Evol. 2006; 23:254–267. doi: 10.1093/molbev/msj030.

62. Pembleton LW, Cogan NOI, Forster JW. StAMPP: an R package for calculation of genetic differentiation and structure of mixed-ploidy level populations. Mol Ecol Resour 2013; 13:946–952. doi: 10.1111/1755-0998.12129.

63. Bolker BM, Brooks ME, Clark CJ, Geange SW, Poulsen JR, Stevens MH, et al. Generalized linear mixed models: a practical guide for ecology and evolution. Trends Ecol Evol. 2009; 24:127–135. doi: 10.1016/j.tree.2008.10.008.

64. Harris T, Yang Z, Hardin JW. Modeling underdispersed count data with generalized Poisson regression. The Stata Journal. 2012; 12:736–747. doi: 10.1177/1536867X1201200412.

65. Huang A. Mean-parametrized Conway–Maxwell–Poisson regression models for dispersed counts. Statistical Modelling. 2017; 17:1–22. doi: 10.1177/1471082X17697749.

66. Harrison XA. A comparison of observation-level random effect and Beta-Binomial models for modelling overdispersion in Binomial data in ecology & evolution. PeerJ. 2015; 3:e1114. doi: 10.7717/peerj.1114.

67. Bolker BM. Ecological models and data in R. Princeton: Princeton University Press; 2008. doi: 10.1515/9781400840908.

68. Lenth RV. Least-squares means: the R package lsmeans. Journal of Statistical Software. 2016; 69:1–33. doi: 10.18637/jss.v069.i01.

69. Nakagawa S, Johnson PC, Schielzeth H. The coefficient of determination R2 and intra-class correlation coefficient from generalized linear mixed-effects models revisited and

expanded. Journal of the Royal Society Interface. 2017; 14:20170213. doi: 10.1098/rsif.2017.0213.

70. Hartig F. DHARMa: Residual diagnostics for hierarchical (multi-level / mixed) regression models. R package version 0.4.6 [software]. 2022. [cited 2023 Sep 28]. Available from: https://cran.r-project.org/web/packages/DHARMa/.

71. Lenth RV. emmeans: estimated marginal means, aka least-squares means. R package version 1.8.4-1 [software]. 2023. [cited 2023 Sep 28]. Available from: https://cran.r-project.org/web/packages/emmeans/.

72. Wickham H. ggplot2: elegant graphics for data analysis. New York: Springer-Verlag; 2016.

73. Brooks ME, Kristensen K, van Benthem KJ, Magnusson A, Berg CW, Nielsen A, et al. GlmmTMB balances speed and flexibility among packages for zero-inflated generalized linear mixed modelling. R J. 2017; 9:378–400. doi: 10.32614/RJ-2017-066.

74. Lüdecke D, Ben-Shachar MS, Patil I, Waggoner P, Makowski D. performance: An R package for assessment, comparison and testing of statistical models. Journal of Open Source Software. 2021; 6:3139. doi: 10.21105/joss.03139.

75. Combs JK, Lambert AM, Reichard SH. Predispersal seed predation is higher in a rare species than in its widespread sympatric congeners (*Astragalus*, Fabaceae). Am J Bot. 2013; 100:2149–2157. doi: 10.3732/ajb.1300238.

76. Ortega-Baes P, Gorostiague P. Extremely reduced sexual reproduction in the clonal cactus *Echinopsis thelegona*. Pl Syst Evol. 2013; 299:785–791. doi: 10.1007/s00606-013-0761-6.

77. Atasagun B, Aksoy A, Güllü IB, Albayrak S. Reproductive biology of *Astragalus argaeus* (Fabaceae), a critically endangered endemic species. An Acad Bras Cienc. 2021; 93:e20201613. doi: 10.1590/0001-3765202120201613.

78. Grzyl A, Kiedrzyński M, Zielińska KM, Rewicz A. The relationship between climatic conditions and generative reproduction of a lowland population of *Pulsatilla vernalis*: the last breath of a relict plant or a fluctuating cycle of regeneration? Plant Ecol. 2014; 215:457–466. doi: 10.1007/s11258-014-0316-0.

79. Bogdziewicz M, Szymkowiak J, Fernández-Martínez M, Penuelas J, Espelta J. The effects of local climate on the correlation between weather and seed production differ in two species with contrasting masting habit. Agric For Meteorol. 2019; 268:109–115 doi: 10.1016/j.agrformet.2019.01.016.

80. Wypych A, Ustrnul Z, Sulikowska A, Chmielewski F-M, Bochenek B. Spatial and temporal variability of the frost-free season in Central Europe and its circulation background. Int J Climatol. 2017; 37:3340–3352. doi: 10.1002/joc.4920.

81. Lawson DA, Rands SA. The effects of rainfall on plant–pollinator interactions. Arthropod Plant Interact. 2019; 13:561–569. doi: 10.1007/s11829-019-09686-z.

82. Alonso C. Early blooming's challenges: extended flowering season, diverse pollinator assemblage and the reproductive success of gynodioecious *Daphne laureola*. Ann Bot. 2004; 93:61–66. doi: 10.1093/aob/mch004.

83. Rodríguez-Pérez J, Travaset A. Influence of reproductive traits on pollination success in two *Daphne* species (Thymelaeaceae). J Plant Res. 2011; 124:277–87. doi: 10.1007/s10265-010-0373-y

84. Liu S, Yang A, Zhou H, Yu F. Reproductive characteristics of *Daphne aurantiaca*. Guihaia. 2018; 38:626–634. doi: 10.11931/guihaia.gxzw201705037.

85. Larson DW, Matthes U, Kelly PE. Cliff ecology: pattern and process in cliff ecosystems. New York: Cambridge University Press; 2000.

86. Scherrer D, Körner C. Topographically controlled thermal habitat differentiation buffers alpine plant diversity against climate warming. J Biogeogr. 2011; 38:406–416. doi: 10.1111/j.1365-2699.2010.02407.x.

87. Carvallo GO, Vergara-Meriño B, Díaz A, Villagra CA. Rocky outcrops conserve genetic diversity and promote regeneration of a threatened relict tree in a critically endangered ecosystem. Biodivers Conserv. 2019; 28:2805–2824. doi: 10.1007/s10531-019-01797-6.

88. Gitzendanner MA, Weekley CW, Germain-Aubrey CC, Soltis DE, Soltis PS. Microsatellite evidence for high clonality and limited genetic diversity in *Ziziphus celata*

(Rhamnaceae), an endangered, self-incompatible shrub endemic to the Lake Wales Ridge, Florida, USA. Conserv Genet. 2012; 13:223–234. doi: 10.1007/s10592-011-0287-9.

89. Silva CA, Vieira MF, Carvalho-Okano RM, Oliveira LO. Reproductive success and genetic diversity of *Psychotria hastisepala* (Rubiaceae), in fragmented Atlantic forest, Southeastearn Brazil. Rev Biol Trop. 2014; 62: 309–319. doi: 10.15517/rbt.v62i1.5854.

90. Petrova G, Petrov S, Möller M. Low genetic diversity in small leading edge populations of the European paleoendemic *Ramonda serbica* (Gesneriaceae) in Bulgaria. Nord J Bot. 2018; 36:njb-01655. doi: 10.1111/njb.01655.

91. Stojanova B, Šurinová M, Zeisek V, Münzbergová Z, Pánková H. Low genetic differentiation despite high fragmentation in the endemic serpentinophyte *Minuartia smejkalii* (*M. verna* agg., Caryophyllaceae) revealed by RADSeq SNP markers. Conserv Genet. 2020; 21:187–198. doi: 10.1007/s10592-019-01239-4.

92. Zhao Y, Yin G, Gong X. RAD-sequencing improves the genetic characterization of a threatened tree peony (*Paeonia ludlowii*) endemic to China: implications for conservation. Plant Divers. 2022; doi: 10.1016/j.pld.2022.07.002.

93. Dang Z, Li J, Liu Y, Song M, Lockhart PJ, Tian Y, et al. RADseq-based population genomic analysis and environmental adaptation of rare and endangered recretohalophyte *Reaumuria trigyna*. Plant Genome. 2023; e20303:1–18. doi: 10.1002/tpg2.20303.

94. Brys R, Jacquemyn H, Endels P, Van Rossum F, Hermy M, Triest L, et al. Reduced reproductive success in small populations of the self-incompatible *Primula vulgaris*. J Ecol. 2004; 92:5–14. doi: 10.1046/j.0022-0477.2004.00840.x.

95. Navascués M, Stoeckel S, Mariette S. Genetic diversity and fitness in small populations of partially asexual, self-incompatible plants. Heredity. 2010; 104:482–492. doi: 10.1038/hdy.2009.159.

96. Calviño-Cancela M, Escudero M, Rodrígeuz-Pérez J, Cano E, Vargas P, Velo-Antón G, et al. The role of seed dispersal, pollination and historical effects on genetic patterns of an insular plant that has lost its only seed disperser. J Biogeogr. 2012; 39:1996–2006. doi: 10.1111/j.1365-2699.2012.02732.x.

97. Vermeersch S, Triest L. Distylic *Hottonia palustris* shows high reproductive success in small populations despite low genetic variability. Aquat Bot. 2006; 84:354–358. doi: 10.1016/j.aquabot.2006.01.004.

98. Plenk K, Bardy K, Höhn M, Kropf M. Long-term survival and successful conservation? Low genetic diversity but no evidence for reduced reproductive success at the north-westernmost range edge of *Poa badensis* (Poaceae) in Central Europe. Biodivers Conserv. 2019; 28:1245–1265. doi: 10.1007/s10531-019-01722-x.

99. Kikuzawa K. Floral biology and evolution of gynodioecism in *Daphne kamtchatica* var. *jezoensis*. Oikos. 1989; 56:196–202. doi: 10.2307/3565336.

100. Alonso C, Herrera CM. Neither vegetative nor reproductive advantages account for high frequency of male-steriles in southern Spanish gynodioecious *Daphne laureola* (Thymelaeaceae). Am J Bot. 2001; 88:1016–1024. doi: 10.2307/2657083.

101. Barbi S. Studi di biologia riproduttiva per la conservazione di alcune specie vegetali dell'ambiente mediterraneo e per la valorizzazione di produzioni eco-compatibili. Ph.D. Thesis, University of Naples Federico II. 2008. Available from: http://www.fedoa.unina.it/3462/1/Tesi_Dottorato_Barbi_Sara.pdf.

102. Roccotiello E, Casazza G, Galli L, Cornara L, Moncalvo A, Minuto L. The flower biology of *Daphne gnidium* L. (Thymelaeaceae). Plant Ecol Evol Syst. 2009; 279:41–49. doi: 10.1007/s00606-009-0144-1.

103. Roccotiello E, Casazza G, Cornara L, Moncalvo A, Minuto L. Reproducitve success in *Daphne gnidium* (Thymelaeaceae). Boll Mus Ist Biol Univ. 2012; 74:22–37.

104. Klimko M. Obserwacje fenologiczne organów generatywnych *Daphne mezereum* L. w rezerwacie „Wydymacz" kolo Antonina. Rocz Ak Rol Poz. 1999; 2:51–66.

105. Oostermeijer JGB, Luijten SH, Petanidou T, Kos M, Ellis-Adam AC, Den Nijs JCM. Pollination in rare plants: is population size important? Det Norske Videnskaps-akademi. I.Matematisk Naturvidenskapelige Klasse, Skrifter, Ny Serie. 2000; 39:201–213.

106. Charlesworth D, Charlesworth B. Quantitative genetics in plants: the effect of the breeding system on genetic variability. Evolution. 1995; 49:911–920. doi: 10.2307/2410413.

107. Šedivá J, Žlebčík J. Vegetative and generative propagation of the endangered species *Daphne cneorum* L. Acta Pruhoniciana. 2010; 96:15–18.

108. Clark-Tapia R, Mandujano MC, Valverde T, Mendoza A. How important is clonal recruitment for population maintenance in rare plant species?: the case of the narrow endemic cactus, *Stenocereus eruca*, in Baja California, México. Biol Conserv. 2003; 124:123–132. doi: 10.1016/j.biocon.2005.01.019.

109. Abrahamson II. WG, Kloet SPV. The reproduction and ecology of *Hypericum edisonianum:* an endangered Florida endemic. Castanea. 2014; 79:168–181. doi: 10.2179/14-016.

110. Herben T, Šerá B, Klimešová J. Clonal growth and sexual reproduction: tradeoffs and environmental constraints. Oikos. 2015; 124:469–76. doi: 10.1111/oik.01692.

Supporting information

S1 Fig. Distribution map of studied populations of *Daphne arbuscula* in the area Muránska planina Mts in the Western Carpathians and distances among the populations.

S2 Table. Overview of the breeding system testing in studied populations of *Daphne arbuscula*.

S3 Table. Reproduction and population-genetic characteristics of the studied individuals of *Daphne arbuscula.*

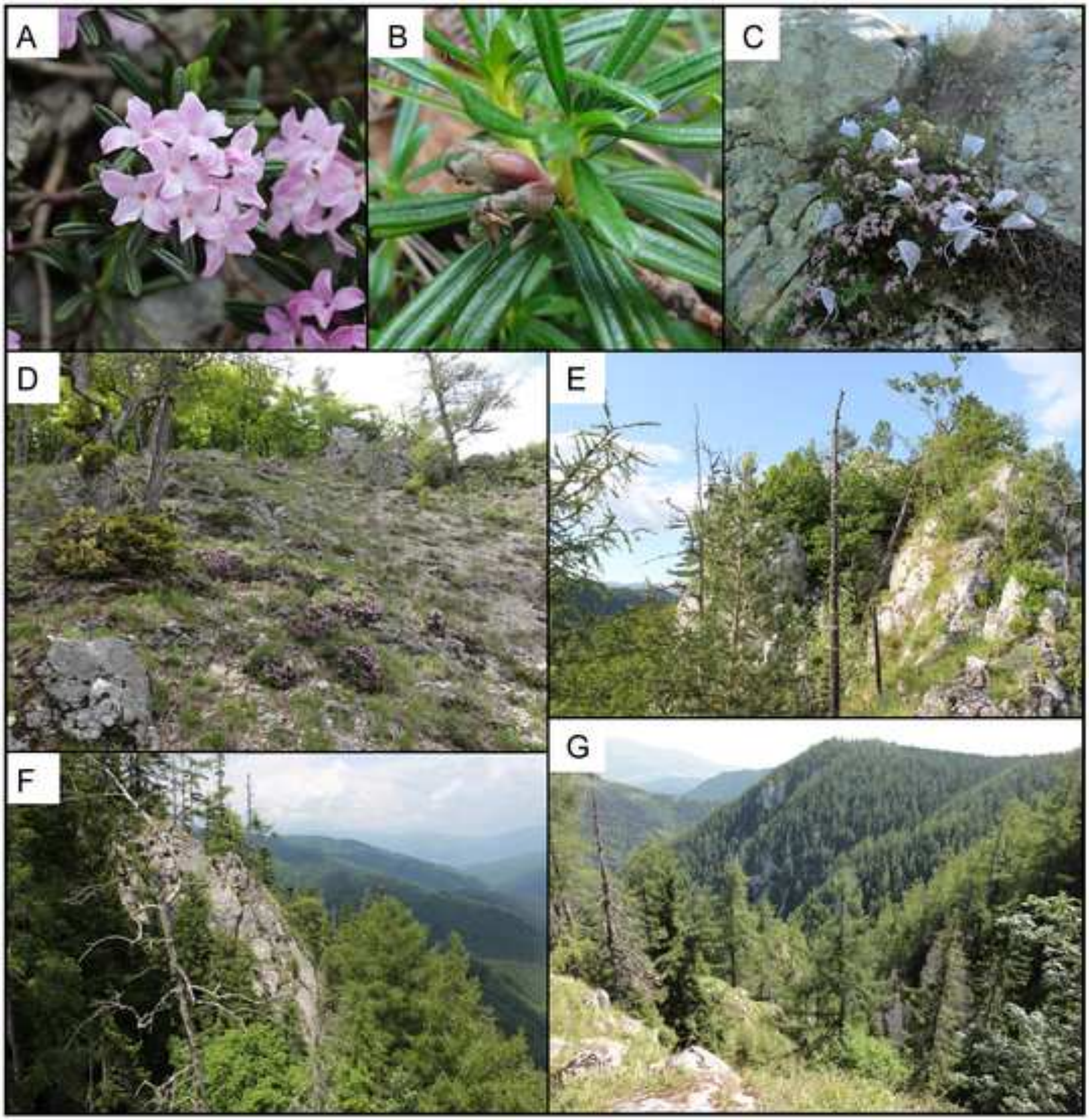
A
B
C
D
E
F
G

Click here to access/download;Figure;Fig2.tif

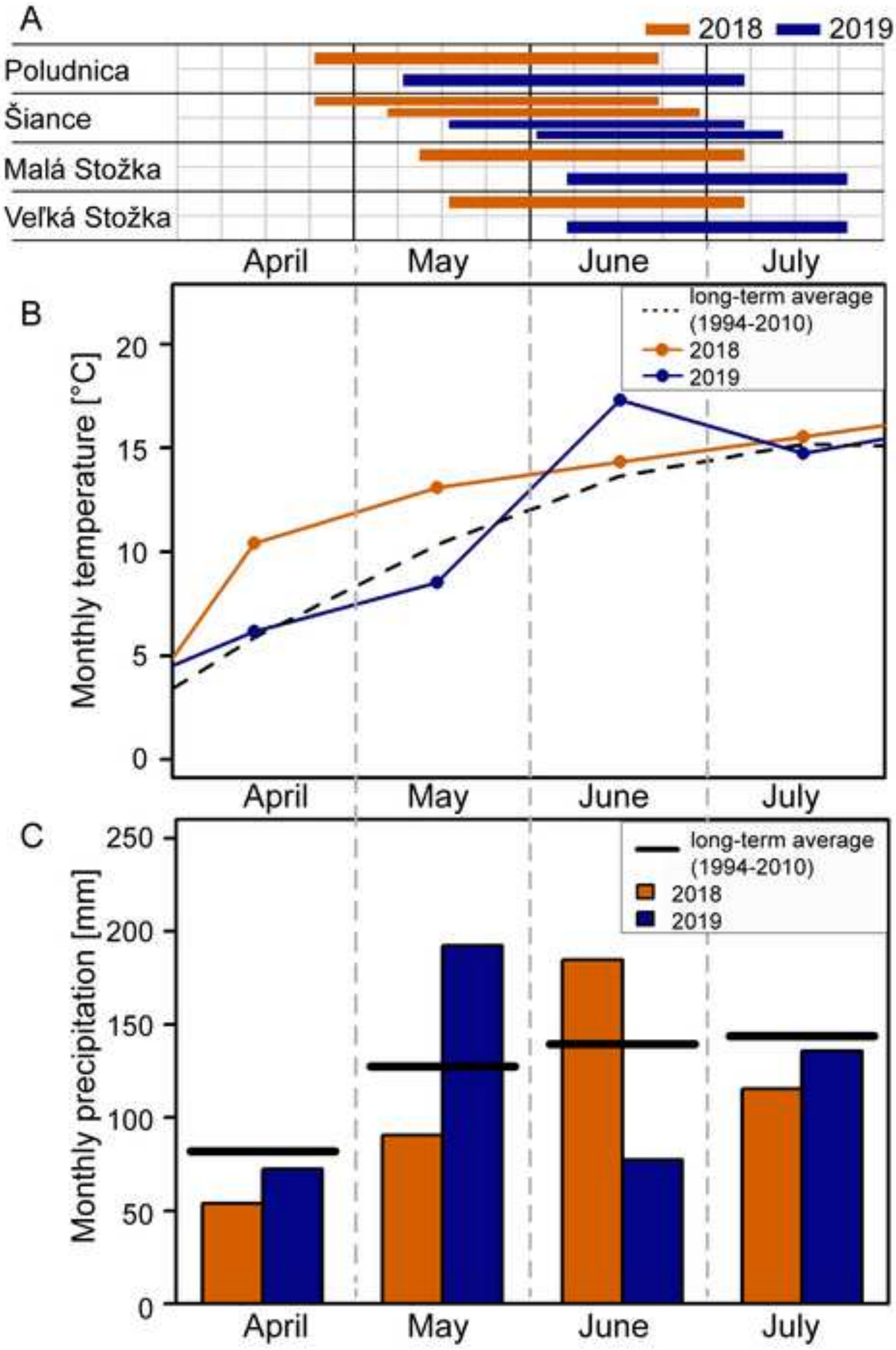

 Click here to access/download;Figure;Fig3.tif

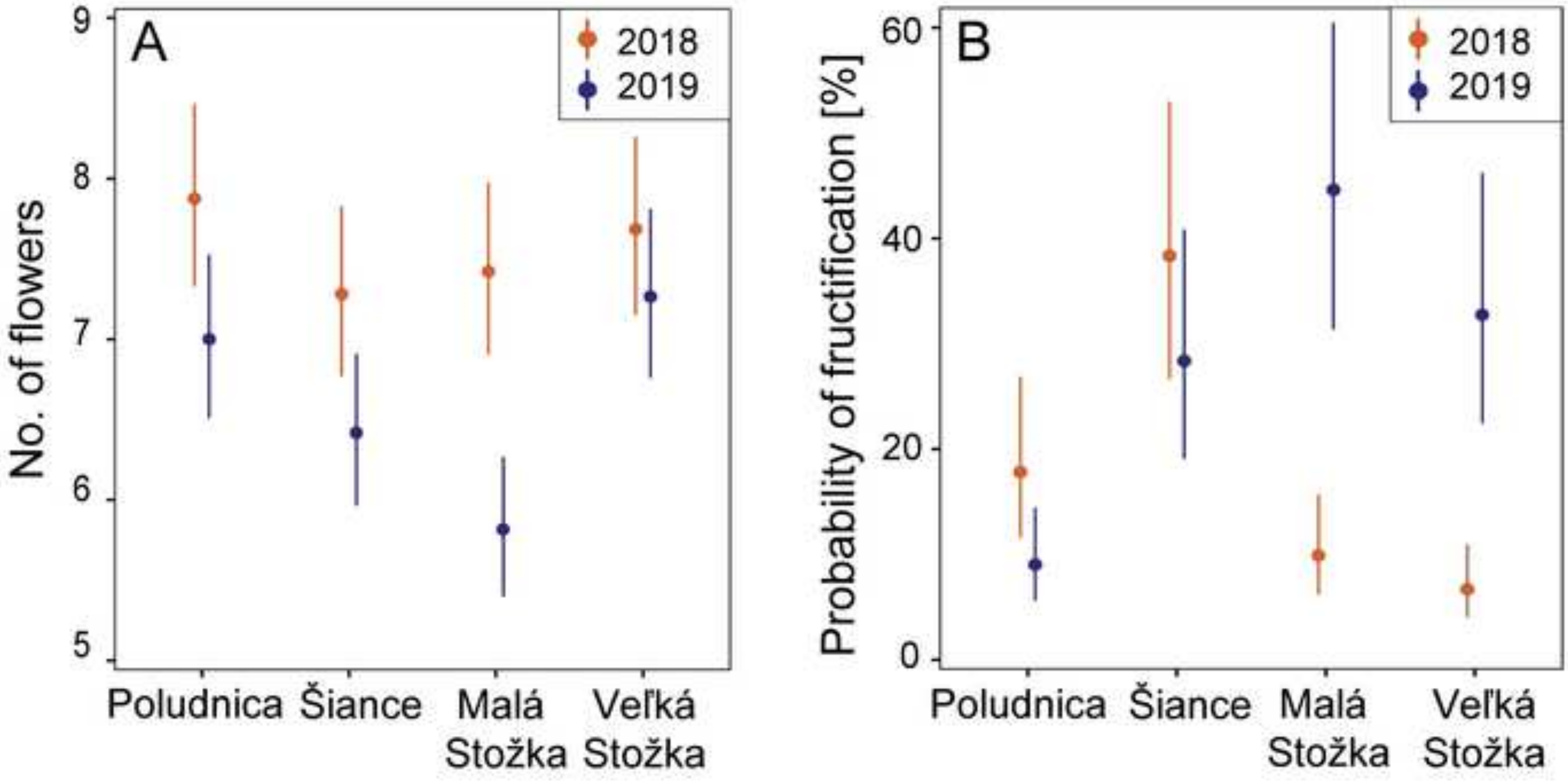

Figure_4_Gajdosova et al_2023

Click here to access/download;Figure;Fig4.tif

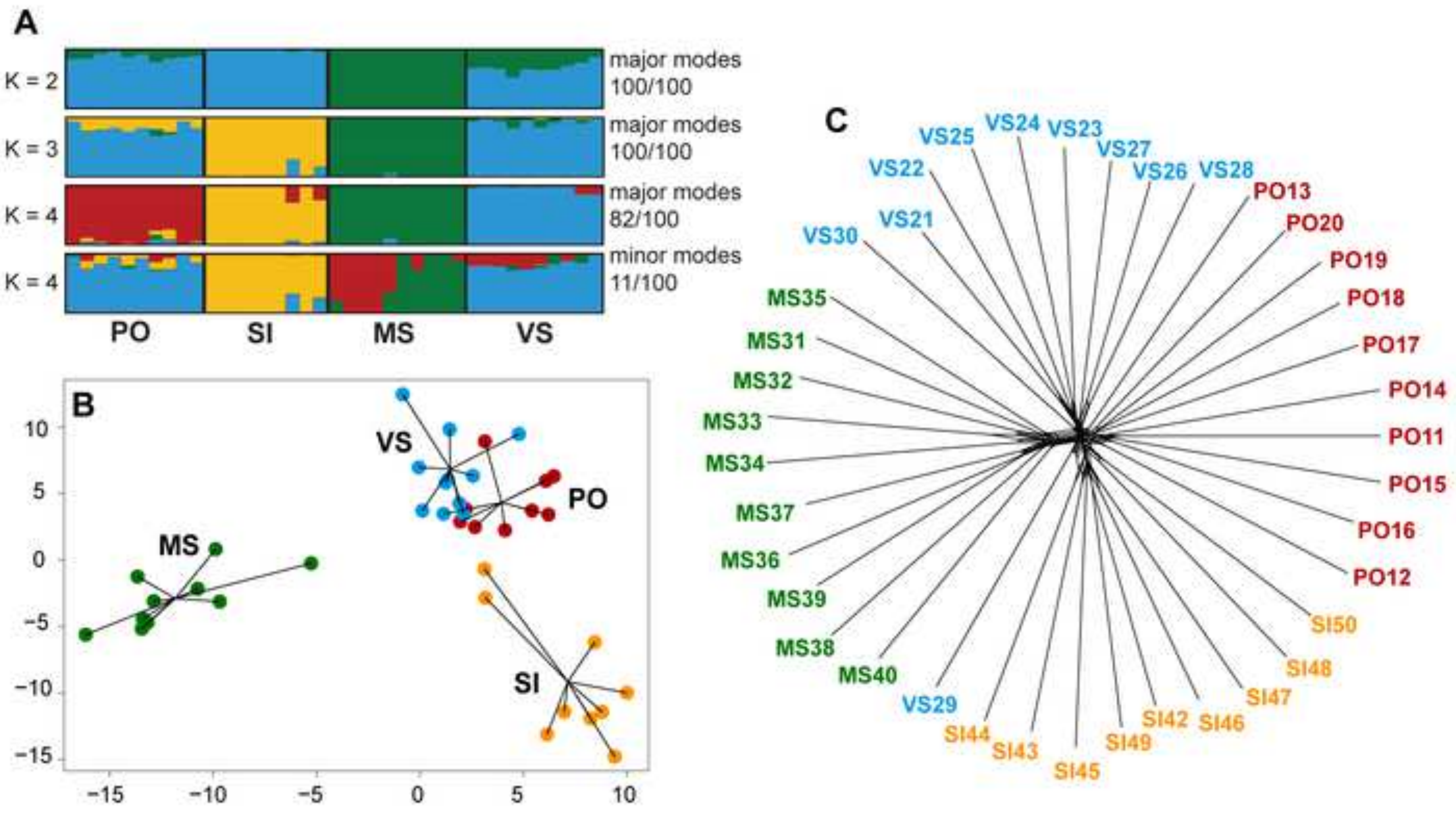

Supplementary data_Fig_S1

Click here to access/download
Supporting Information
S1_Figure.pdf

Supplementary data_Table_S2

Click here to access/download
Supporting Information
S2_Table.pdf

Supplementary data_Table_S3

Click here to access/download
Supporting Information
S3_Table.pdf
