# Supplementary material for: Unravelling some factors affecting sexual reproduction in rock-specialist shrub: Insight from an endemic Daphne arbuscula (Thymelaeaceae)
Source: PLoS One. 2024 May 9;19(5):e0300819. doi: 10.1371/journal.pone.0300819 (PMC11081377; doi:10.1371/journal.pone.0300819)
Supplement: Attachment — Submitted filename: Response to Reviewers_R2_Gajdosova et al. 2024.docx [file pone.0300819.s005.docx]

**PONE-D-23-32886R1**

**Unravelling some factors affecting sexual reproduction in rock-specialist shrub: insight from an endemic Daphne arbuscula (Thymelaeaceae)**

**Dear Editors,**

**We highly appreciate the second round of insightful feedback from both reviewers and the editor. All suggested changes have been incorporated into the revised manuscript, presented in a point-by-point response below, marked in bold. The tracked changes reflect these modifications. All figures were controlled by the PACE software, ensuring compliance with PLOS ONE guidelines.**

**Sincerely,**

**Marek Slovak**

**Journal Requirements:**

Please review your reference list to ensure that it is complete and correct. If you have cited papers that have been retracted, please include the rationale for doing so in the manuscript text, or remove these references and replace them with relevant current references. Any changes to the reference list should be mentioned in the rebuttal letter that accompanies your revised manuscript. If you need to cite a retracted article, indicate the article’s retracted status in the References list and also include a citation and full reference for the retraction notice.

**We checked all citations and the reference list.**

**Review Comments to the Author:**

**Reviewer #1:**

I appreciate the great effort you have made to revise this manuscript. Below are some suggestions for improvement, which I think should be taken into consideration:

Introduction/Figure 1: It is somewhat confusing that the results of the molecular analyses are already shown in Figure 1. I would separate Figure 1A from the other panels. 1A is then shown as a separate figure in the Introduction and 1B-D in the results (because this a major part of your results and discussion!). Since the revision means that there are only two figures in the main text, I see no problem in showing three figures. Alternatively, Figure 1 could also be mentioned in the M&M. I would reformulate lines 96-100 e.g.: “In this study we investigated four populations of D. arbuscula, although limited to one mountain system (Muránska planina Mts in the Western Carpathians, Slovakia), demonstrate significant niche diversity, specifically those two residing at lower altitudes and facing south have typically drier and warmer climatic conditions, whereas those two populations located at higher altitudes on the northern slopes experience harsher and wetter conditions with a montane microclimate [19,20].”

**Thank you for your continued efforts in revising our manuscript. We appreciate your valuable proposals and recommendations. We have implemented the suggested changesas follows:**

1) **We followed your suggestion and split Figure 1 into Figs. 1 and 3. Figure 1 (former Figure 1A) now includes only the distribution map and is placed in the Materials and Methods section. The formed Figures 1B-D, containing genetic analyses, have been relocated to the Results section and are now presented as Figs 3A-C**
2) L330: “populations” not “population”.

**Corrected (p. 14 lns. 323)**

**Reviewer #2:**

Dear Authors, thank you for addressing my comments. I only have a few minor edits that can be addressed during the proofing of the manuscript. It was nice to learn about your research and the conservation work associated with endemic Daphne arbuscula (Thymelaeaceae).

**Thank you for your additional valuable comments and edits. We implemented them in the manuscript:**

**1) Following words were reworded accordingly:**

**p. 7 lns. 157-159 and 172;**

**p. 9 lns. 207, 210, 214, 219;**

**p. 13 lns. 310, 313;**

**p. 19 lns. 434**

2) Deleted heading “Statistical analysis” in M&M section

**Deleted.**

3) This is not a heading, is a statement Perhaps change to "Role of genetic variability on sexual reproduction"

**Reworded heading in the Discussion section: Done as recommended (p. 21 lns. 495-496): „Low association between genetic variability at the population level and sexual reproduction in *D. arbuscula*“**

4) Correction in the financial disclosure (typo) - updated statement:

**Done.**
